# Crystal structure from X-ray powder diffraction data, DFT-D calculation, Hirshfeld surface analysis, and energy frameworks of (*RS*)-trichlorme­thia­zide

**DOI:** 10.1107/S2056989021013633

**Published:** 2022-01-07

**Authors:** Robert A. Toro, Analio Dugarte-Dugarte, Jacco van de Streek, José Antonio Henao, José Miguel Delgado, Graciela Díaz de Delgado

**Affiliations:** a Grupo de Investigación en Química Estructural (GIQUE), Escuela de Química, Facultad de Ciencias, Universidad Industrial de Santander, Bucaramanga, Colombia; b Laboratorio de Cristalografía-LNDRX, Departamento de Química, Facultad de Ciencias, Universidad de los Andes, Mérida 5101, Venezuela; c Avant-garde Materials Simulation, Alte Str. 2, D-79249 Merzhausen, Germany

**Keywords:** trichlorme­thia­zide, structure determination, powder diffraction, DFT-D calculations, Hirshfeld surface analysis, energy frameworks

## Abstract

The structure of racemic (*RS*)-trichlorme­thia­zide [systematic name: (*RS*)-6-chloro-3-(di­chloro­meth­yl)-1,1-dioxo-3,4-di­hydro-2*H*-1λ^6^,2,4-benzo­thia­di­azine-7-sulfonamide], C_8_H_8_Cl_3_N_3_O_4_S_2_ (*RS*-TCMZ), a diuretic drug used in the treatment of oedema and hypertension, was determined from laboratory X-ray powder diffraction data using *DASH* [David *et al.* (2006 ▸). *J. Appl. Cryst.* **39**, 910–915.], refined by the Rietveld method with *TOPAS-Academic* [Coelho (2018 ▸). *J. Appl. Cryst.* **51**, 210–218], and optimized using DFT-D calculations. The extended structure consists of head-to-tail dimers connected by π–π inter­actions which, in turn, are connected by C—Cl⋯π inter­actions. They form chains propagating along [101], further connected by N—H⋯O hydrogen bonds to produce layers parallel to the *ac* plane that stack along the *b*-axis direction, connected by additional N—H⋯O hydrogen bonds. The Hirshfeld surface analysis indicates a major contribution of H⋯O and H⋯Cl inter­actions (32.2 and 21.7%, respectively). Energy framework calculations confirm the major contribution of electrostatic inter­actions (*E*_elec_) to the total energy (*E*_tot_). A comparison with the structure of *S*-TCMZ is also presented.

## Abstract 2

The structure of the racemic form of the diuretic drug trichlorme­thia­zide was determined from laboratory X-ray powder diffraction data: the extended structure features an intricate combination of N—H⋯O hydrogen bonds and π–π and C—Cl⋯π inter­actions.

## Chemical context

Trichlorme­thia­zide (TCMZ), systematic name 6-chloro-3-(di­chloro­meth­yl)-1,1-dioxo-3,4-di­hydro-2*H*-1λ^6^,2,4-benzo­thia­di­azine-7-sulfonamide (C_8_H_8_Cl_3_N_3_O_4_S_2_), is a diuretic drug derived from thia­zide, the precursor of a classic family of diuretic compounds, discovered in the 1950s. The first approved drug of this class, chloro­thia­zide, was marketed under the trade name Diuril in 1958 (Beyer, 1993 ▸). The compound under study, trichlorme­thia­zide, has a similar chemical structure to hydro­chloro­thia­zide, the most prescribed member of the group (Hripcsak *et al.*, 2020 ▸). The difference is the substitution of one hydrogen atom of the methyl­ene group by a CHCl_2_ di­chloro­methyl group. Thia­zide diuretics and their derivatives are primarily used in the treatment of mild to moderate hypertension and oedema associated with Na^+^ and K^+^ retention and expansion of the extracellular fluid volume. They also increase Ca^2+^ excretion, a potentially useful effect in patients with hypercalciuria, a condition that could lead to the formation of kidney stones (Menè, 2004 ▸). It is commonly used around the world under different brand names such as Achletin, Aqua­cot, Diu-hydrin, Diurese, Metahydrin, Naqua, Triflumen, as well as with the generic trichlorme­thia­zide name. Given our inter­est in the structure of materials involved in pharmaceutical formulations or with potential pharmaceutical applications, it was decided to undertake the structure determination of the racemic form of this active pharmaceutical ingredient (API). 
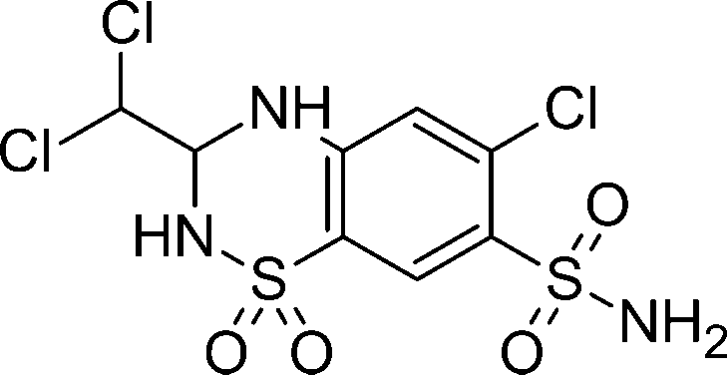


## Structural commentary

The refinement of the final structural model using powder diffraction data recorded showed bond distances and angles within the range suggested in the statistical analysis performed with the *Mogul* geometry check (Bruno *et al.*, 2004 ▸). Only two out of 56 distances and bond angles in the structure are classified in the analysis as ‘unusual’. However, these two ‘unusual’ parameters are close to the values suggested by the *Mogul* geometry analysis, with *Z*-scores below 3. These parameters are similar to the values reported for the *S*-isomer (Cambridge Structural Database refcode KIKCUD; Fernandes *et al.*, 2007 ▸) and for the DFT-D-optimized racemic structure.

**Figure 1:**
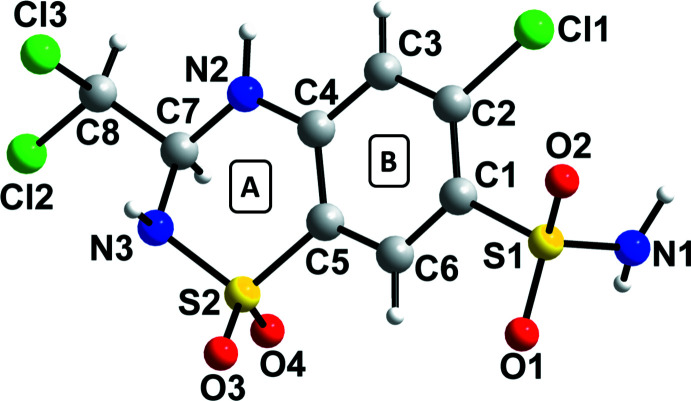
Mol­ecular structure of *RS*-TCMZ with the atom- and ring-labelling scheme.

The asymmetric unit contains one TCMZ mol­ecule (Fig. 1 ▸): the stereogenic centre C7 has an *S* configuration but crystal symmetry generates a racemic mixture. The thia­zide ring (*A*) exhibits a conformation that could be described as distorted half-chair to distorted envelope at N3 (Spek, 2020 ▸). The substituents in the ring are in bis­ecting (S1—O3), axial (S1—O4, N3—H3*A*, C7—H7) and equatorial (C7—C8, C4—C3, C5—C6, N2—H2*A*) conformations. The almost planar benzosulfonamide ring (ring *B*) makes an angle of 8.2 (2)° with the best plane through the thia­zide ring. The mol­ecule is oriented almost parallel to the *a*-axis as indicated by a 3.11 (8)° angle (*PLATON*; Spek, 2020 ▸), which corresponds to the angle between the *a*-axis and the perpendicular to the normal of the best least-square plane defined by the atoms of the two rings. The angle between the corresponding *A* and *B* rings in *S*-TCMZ is smaller than in *RS*-TCMZ [4.7 (2)°].

**Figure 2:**
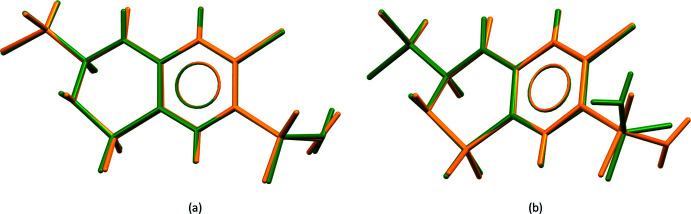
Superposition of the *S*-mol­ecule in racemic TCMZ (yellow) with the mol­ecule in the *S*-TCMZ study (green) reported in KIKCUD (*a*) allowing flexibility and (*b*) no flexibility allowed.

Fig. 2 ▸ shows a superposition of the mol­ecule with an *S*-configuration in racemic TCMZ with the mol­ecule of the *S*-enanti­omer in KIKCUD. When flexibility is allowed in the superposition (Fig. 2 ▸ *a*), the r.m.s.d. deviation is 0.070 and the maximum deviation (max. D) is 0.146 Å. Without flexibility, the values for r.m.s.d. and max. D are 0.785 and 2.763 Å, respectively (Fig. 2 ▸ *b*). The difference between the two conformations lies in the orientation of the sulfonamide group and leads to differences in the hydrogen-bonding patterns between the two compounds as discussed below.

***Intra­molecular hydrogen bonds***

**Figure 3:**
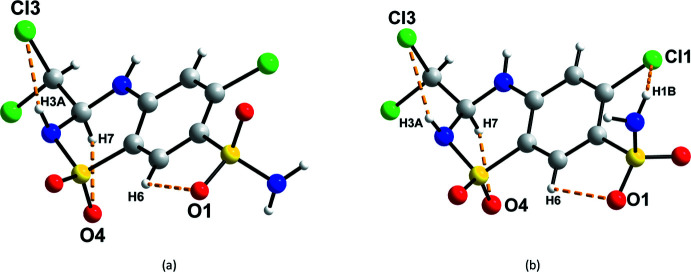
Intra­molecular hydrogen bonds present in (*a*) *RS*-TCMZ and (*b*) *S*-TCMZ (KIKCUD).

Three different intra­molecular hydrogen bonds are present in *RS*-TCMZ (Fig. 3 ▸ *a*, Table 1 ▸). The shortest contact involves C6—H6⋯O1 with an H⋯*A* distance of 2.372 (8) Å and a *D*—H⋯*A* angle of 106.9 (5)°. A second intra­molecular hydrogen bond occurs between C7—H7 and O4 [2.584 (10) Å, 106.1 (5)°]. The third contact, N3—H3*A*⋯Cl3, has geometric parameters 2.767 (8) Å and 102.1 (5)°. The three hydrogen bonds can all be represented by the graph-set symbol *S*(5) (Etter *et al.*, 1990 ▸; Bernstein *et al.*, 1995 ▸). The *S*-isomer displays the same intra­molecular hydrogen bonds observed in *RS*-TCMZ. However, as a result of the orientation of the –NH_2_ group, an additional intra­molecular inter­action between N1—H1*B* and Cl1 is possible in *S*-TCMZ (Fig. 3 ▸ *b*).

## Supra­molecular features

***Inter­molecular hydrogen bonds***

**Figure 4:**
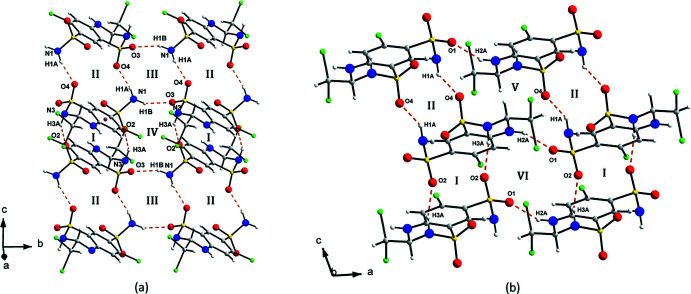
(*a*) Graph-set analysis of the inter­molecular N—H⋯O hydrogen bonds in *RS*-TCMZ projected onto the *bc* plane. (*b*) Sequence of hydrogen-bond motifs viewed down the *b* axis. The hydrogen-bonded motifs are labelled as **I**: [ 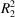 (16)]^O2^_N3_; **II**: [ 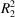 (16)]^O4^_N1_; **III**: [ 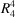 (12)]^O3,O4^_N1,N1_; **IV**: [ 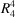 (16)]^O2,O3^_N1,N3_; **V**: [ 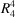 (20)]^O1,O4^_N2,N1_; **VI**: [ 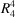 (16)]^O1,O2^_N2,N3_.

Given the number of potential hydrogen-bond donors and acceptors, the hydrogen-bonding pattern in *RS*-TCMZ is very rich and relevant geometric parameters are summarized in Table 1 ▸. Fig. 4 ▸ depicts selected views of the inter­molecular hydrogen bonds present in racemic TCMZ where the O and H atoms involved in hydrogen bonds are labelled. As shown in Fig. 4 ▸ *a*, an 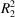 (16) loop, with O2 as the acceptor, N3 as the donor, and H3*A* as the donated H ([ 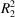 (16)]^O2^_N3_, motif **I**) alternate with a motif [ 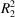 (16)]^O4^_N1_ (H1*A*, motif **II**), forming tapes propagating along the *c*-axis direction. These tapes are joined by a sequence of [ 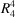 (12)]^O3,O4^_N1,N1_ (H1*A*, H1*B*, **III**) and [ 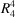 (16)]^O2,O3^_N1,N3_ (H1*B*, H3*A*, **IV**) motifs, resulting in layers lying parallel to the *bc* plane. Perpendicular to these layers (Fig. 4 ▸ *b*) the **I**/**II** motifs are connected by [ 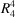 (20)]^O1,O4^_N2,N1_ (H2*A*, H1*A*, **V**) and [ 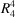 (16)]^O1,O2^_N2,N3_ (H2*A*, H3*A*, **VI**) motifs, forming layers parallel to the *ac* plane, resulting in an intricate three-dimensional hydrogen-bonded network. In the structure of *S*-TCMZ, N1 and N3 are also involved in hydrogen bonds based on the N—H⋯O heterosynthon. However, N2 participates in the homosynthon N2—H2⋯N1. A C8—H8⋯O3 hydrogen bond is also important in the packing arrangement of *S*-TCMZ.

***π–π and C—Cl⋯π inter­actions***

**Figure 5:**
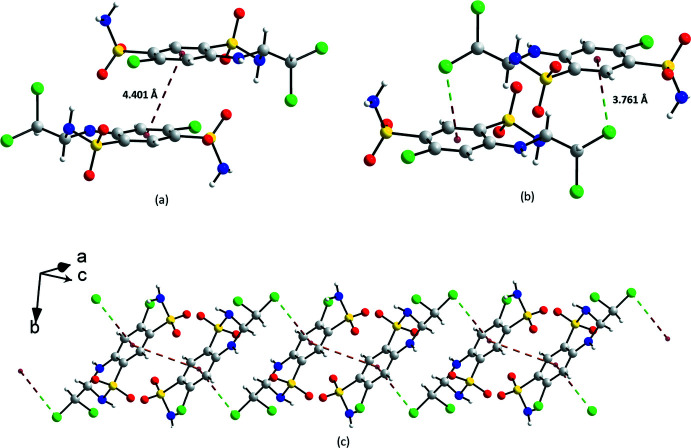
Geometry of (*a*) π–π and (*b*) C—Cl⋯π inter­actions in *RS*-TCMZ; (*c*) sequence of head-to-tail π–π connected dimers and head-to-tail C—Cl⋯π connected dimers in *RS*-TCMZ.

In addition, π–π and C—Cl⋯π inter­actions (Spek, 2020 ▸) provide connectivity between the mol­ecules (Fig. 5 ▸). Short π–π inter­actions [*d* = 4.401 (3) Å], occur between mol­ecules related by the symmetry operation −*x*, 2 − *y*, −*z* (Fig. 5 ▸ *a*). At the same time, the original mol­ecule inter­acts *via* a C—Cl⋯π contact of 3.761 (5) Å with another mol­ecule related by symmetry operation 1 − *x*, 2 − *y*, 1 − *z* (Fig. 5 ▸ *b*). The mol­ecules are arranged as head-to-tail dimers producing chains along [101] as depicted in Fig. 5 ▸ *c*. In contrast, in the structure of *S*-TCMZ only C—Cl⋯π inter­actions are observed (Table 1 ▸). This contact is shorter [*d* = 3.456 (2) Å] than in *RS*-TCMZ.

**Figure 6:**
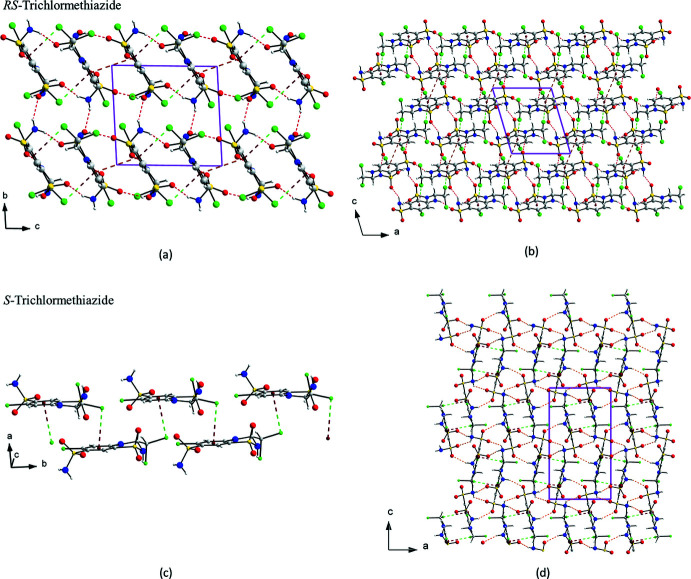
Packing arrangement of *RS*-TCMZ viewed (*a*) down the *a* axis and (*b*) down the *b* axis; (c) chains of *S*-TCMZ mol­ecules connected by C—Cl⋯π inter­actions along the *b* axis; (*d*) view down the *b* axis of chains connected by N—H⋯O and N—H⋯N hydrogen bonds in *S*-TCMZ.

The structure of *RS*-TCMZ is a complex arrangement of hydrogen bonds, π–π and C—Cl⋯π inter­actions as shown in Fig. *6a* and 6*b*. It can be described in terms of chains of head-to-tail dimers connected by π–π inter­actions, which are further connected *via* C—Cl⋯π inter­actions, also in a head-to-tail fashion. These chains are connected by N—H⋯O hydrogen bonds, producing layers parallel to the *ac* plane. The layers stack along the *b*-axis, connected by other N—H⋯O hydrogen bonds. In contrast, the structure of *S*-TCMZ can be described as chains of *S*-TCMZ mol­ecules connected by C—Cl⋯π inter­actions (Fig. 6 ▸ *c*), which form columns along the *b-*axis. These columns are further connected by N—H⋯O and N—H⋯N hydrogen bonds (Fig. 6 ▸ *d*).

## Database survey

A search in the Cambridge Structural Database (CSD, version 5.42, November 2020, updated September 2021; Groom *et al.*, 2016 ▸) by name and by mol­ecular diagram resulted in only one entry. It corresponds to *S*-trichlorme­thia­zide (refcode: KIKCUD; Fernandes *et al.*, 2007 ▸), which reports the structure determined using single-crystal X-ray diffraction data. This enanti­omer crystallizes in the ortho­rhom­bic space group *P*2_1_2_1_2_1_. The PDF-4/Organics database of the Powder Diffraction File (Gates-Rector & Blanton, 2019 ▸) contains two entries associated with this material, PDF 02-094-5865 and PDF 00-039-1828. The first report consists of a calculated pattern based on the CSD report described above. The PDF 00-039-1828 entry contains an experimental pattern with no structural information. The superposition of the recorded pattern and the simulated pattern contained in entry PDF 00-039-1828 (depicted in Fig. S1 of the additional supporting information) shows that they correspond to the same phase. It is worth mentioning that a broader search of the CSD resulted in 100 structures related to TCMZ, among them chloro­thia­zide and hydro­chloro­thia­zide, their polymorphs, derivatives, solvates, and co-crystals.

## Synthesis and crystallization

(*RS*)-Trichlorme­thia­zide was kindly provided by Tecnoquímicas (Cali, Colombia). Based on the FT–IR spectra and the quality of the preliminary diffraction patterns, the present study was carried out on the sample as it was received. Crystallization experiments in different solvents, in search of possible polymorphs, are underway in our laboratories.

## Refinement and DFT-D calculations

**Figure 7:**
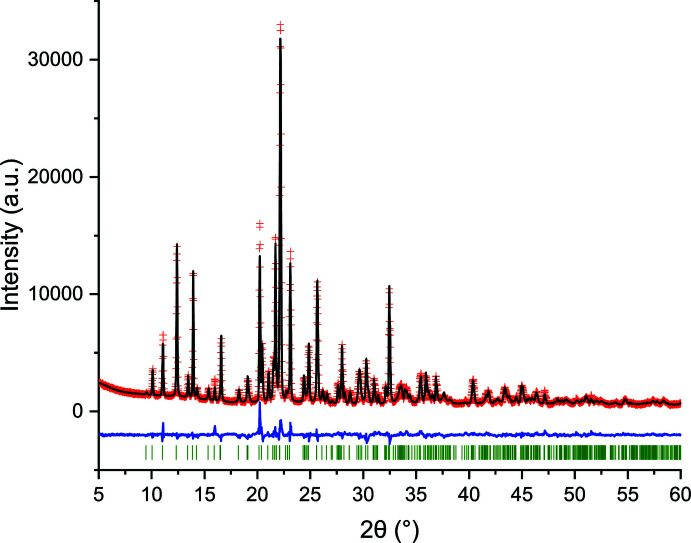
Rietveld plot obtained after the structure refinement of *RS*-TCMZ

Crystal data, data collection and structure refinement details are summarized in Table 2 ▸. The powder diffraction pattern recorded (Fig. 7 ▸) was indexed with *DICVOL14* (Louër & Boultif, 2014 ▸) using the first 30 peaks, producing a triclinic cell with *a* = 8.431 (1) Å, *b* = 8.8919 (9) Å, *c* = 9.720 (2) Å, α = 91.30 (1)°, β = 106.07 (2)°, γ = 97.19 (1)°, *V* = 693.4 (2) Å^3^. The de Wolf (de Wolf, 1968 ▸) and Smith–Snyder (Smith & Snyder, 1979 ▸) figures of merit obtained were *M*_30_ = 53.5 and *F*_30_ = 152.3 (0.0036, 55), respectively. A reduced-cell search in the CSD (Groom *et al.*, 2016 ▸) combined with the chemical elements search having only C, H, N, O, Cl and S yielded no hits.

The fitting of the pattern was carried out with the Pawley algorithm by modelling the background, zero-point and sample displacement errors, cell parameters, peak shape parameters (including anisotropic broadening) using *TOPAS-Academic* (Coelho, 2018 ▸). A 20-term Chebyshev polynomial was used to model the background. The inter­mediate Gaussian–Lorentzian function was employed with a correction for axial divergence as proposed by the program. The Pawley refinement produced a good fit with residuals *R_p_* = 0.02480, *R_wp_* = 0.03280%, and *GoF* = 1.343, strongly supporting the correctness of the unit cell: all the diffraction maxima recorded were accounted for by the triclinic unit cell obtained with *DICVOL14* (Louër & Boultif, 2014 ▸). The initial mol­ecular model, introduced as a ‘.mol’ file, was obtained from the CIF of KIKCUD. With this model and the parameters obtained from the Pawley fit, the crystal structure was determined with *DASH 3.4.5* (David *et al.*, 2006 ▸). The refinement of the structure, carried out with *TOPAS-Academic* (Coelho, 2018 ▸), produced a reasonably good fitting with residuals *R_p_* = 0.0687, *R_wp_* = 0.0931, and *GoF* = 3.985. However, there were discrepancies between the calculated and measured intensity for a few of the most intense diffraction maxima of the pattern.

A DFT-D optimization of this structure, carried out with *GRACE* (Neumann, 2019 ▸), led to a root-mean-square Cartesian displacement (RMSCD) of 0.539 Å. This value is beyond the limit of 0.35 Å considered acceptable for correct structures determined from powder diffraction data (van de Streek & Neumann, 2014 ▸). The examination of the structure with *Mercury* (Macrae *et al.*, 2020 ▸) showed that the structure displayed short N—H⋯Cl—C contacts. The charge distribution on a C—Cl bond is such that at the ‘tip’ (the other side of the atom away from the bond to the carbon atom), the Cl atom has a positive charge. Surprisingly, a hydrogen-bond donor points to this region of the Cl atom. There are several O⋯O contacts around 3.0 Å, which are possible, but surprising given the presence of four N—H hydrogen bond donors. By rotating the O—S(O)—N group around the C1—S1 bond axis by 120° all these inconsistencies disappeared and, therefore, this model was adopted.

The refinement performed with *TOPAS-Academic* (Coelho, 2018 ▸), using the energy-minimized structure as the starting model, was very stable and proceeded smoothly. Fig. 7 ▸ shows the final Rietveld refinement plot. The refinement included an overall scale parameter, the background, the peak shapes (including anisotropic broadening), unit-cell parameters, atomic coordinates and, initially, an overall *B*_iso_ parameter. The bond distances and angles were restrained based on the values of the energy-minimized structure. A planar restraint for the mol­ecule with a standard deviation of 0.01 Å was also established for atoms C1–C6/Cl1/S1. The positions of the hydrogen atoms were refined with restrictions on bond lengths and angles to the atoms to which they are attached, as in the related hydro­chloro­thia­zide form II structure (Florence *et al.*, 2005 ▸). The standard uncertainties of the hydrogen atoms, calculated by *TOPAS*, reflect the propagation of statistical errors from the raw data and do not reflect contributions from systematic errors. More realistic values are somewhat larger than those reported. The isotropic atomic displacement parameters for S and Cl were constrained to be equal and those for C, N, and O were also constrained to be equal. For the hydrogen atoms, they were 1.2 times the *U*_iso_ of the C or N atom to which they are attached.

In total, 177 parameters were refined against 3922 data points, 48 restraints and 2 constraints. The final whole pattern fitting converged with good figures of merit: *R_e_* = 0.02577, *R_p_* = 0.0512, *R_wp_* = 0.0694, *R_B_* = 0.0397, and *GoF* = 2.704. Table 2 ▸ shows the crystal data, experimental parameters, and the refinement parameters obtained. The DFT-D calculations of the new model led to an RMSCD of 0.126 Å, which is lower than the 0.35 Å value (van de Streek & Neumann, 2014 ▸), indicating that the structure determined can be assumed to be correct.

## Computational studies

***Hydrogen-bond propensity analysis***

As several donor and acceptor groups are present in trichlorme­thia­zide, which could form different hydrogen bonding schemes, it was considered of inter­est to carry out a hydrogen-bond propensity (HBP) analysis for this mol­ecule. The HBP analysis was carried out with *Mercury* (Macrae *et al.*, 2020 ▸) for *RS*-TCMZ and for the *S*-TCMZ enanti­omer using data from the CSD entry KIKCUD.

The HBP tool provides an insight into the expected intra- and inter­molecular hydrogen bonds in the structures. For the analysis, the donor atoms considered were N1 (sulfonamide), N2 (secondary amine) and N3 (next to the sulfonyl group). The acceptors were Cl1 (aryl chloride), Cl2/Cl3 (alkyl chloride), N2 (secondary amine), O1/O2 (sulfonamide), and O3/O4 (sulfon­yl). The area under the receiver operating characteristics (ROC) curve was 0.863, indicating good statistical discrimination in the analysis. The results of the calculations are presented in the supporting information.

The intra­molecular hydrogen bond with the highest propensity is N1—H1*B*⋯Cl1 (0.60). This hydrogen bond is observed only in the *S*-enanti­omer. The intra­molecular inter­action involving N3—H3*A*⋯Cl3, observed in the two structures, has the second highest propensity value (0.48).

Regarding the inter­molecular inter­actions, two hydrogen bonds involving the hydrogen atoms bonded to the nitro­gen of the sulfonamide group and the two oxygen atoms of the sulfonyl group (N1—H1*B*⋯O3 and N1—H1*A*⋯O4) have the highest propensities (0.69). They are present in the structure of *RS*-TCMZ (motifs **II** and **III**). However, only one of them (N1—H1*A*⋯O4) is present in the *S*-enanti­omer. The next two inter­actions with highest propensities (0.68) are between the H and O atoms of the sulfonamide groups of two neighboring mol­ecules. One of them (N1—H1*B*⋯O2) is observed only in the *S*-enanti­omer.

The CSD statistics predicts hydrogen bonds for the sulfonyl nitro­gen atom (N3—H3*A*) and for the secondary amine (N2—H2*A*) with the sulfonyl O atoms (propensity values are 0.44 and 0.42, respectively), which are not present in either structure. However, N3—H3*A*⋯O1 and N3—H3*A*⋯O2 contacts with 0.42 propensities are displayed in *S*-TCMZ and *RS*-TCMZ (motifs **I** and **IV**), respectively. In addition, the hydrogen bond N2—H2*A*⋯O1 is present in *RS*-TCMZ (motifs **V** and **VI**) but not in *S*-TCMZ. The hydrogen bond N2—H2*A*⋯N1 was not predicted because the N1 atom was not considered an acceptor. The hydrogen-bond patterns found in the two structures are consistent with the hydrogen-bond propensity analysis results. Every donor and acceptor in *RS*-TCMZ and in *S*-TCMZ has a hydrogen-bond coordination with a high likelihood. Figure S2 of the additional supporting information shows the putative landscape for trichlorme­thia­zide. The two structures fall in the high propensity and hydrogen-bond coordination zone.

***Hirshfeld surface analysis and energy frameworks***

The software *CrystalExplorer21* (Spackman *et al.*, 2021 ▸) was used to produce fingerprint plots of the inter­molecular inter­actions occurring in *RS*-TCMZ and in CSD entry KIKCUD. The parameter *d*_norm_, mapped onto the Hirshfeld surface (Spackman & Jayatilaka, 2009 ▸) is useful to visualize the atoms involved in inter­molecular contacts and the strength of such contacts. Energy frameworks were also calculated with *CrystalExplorer21*.

**Figure 8:**
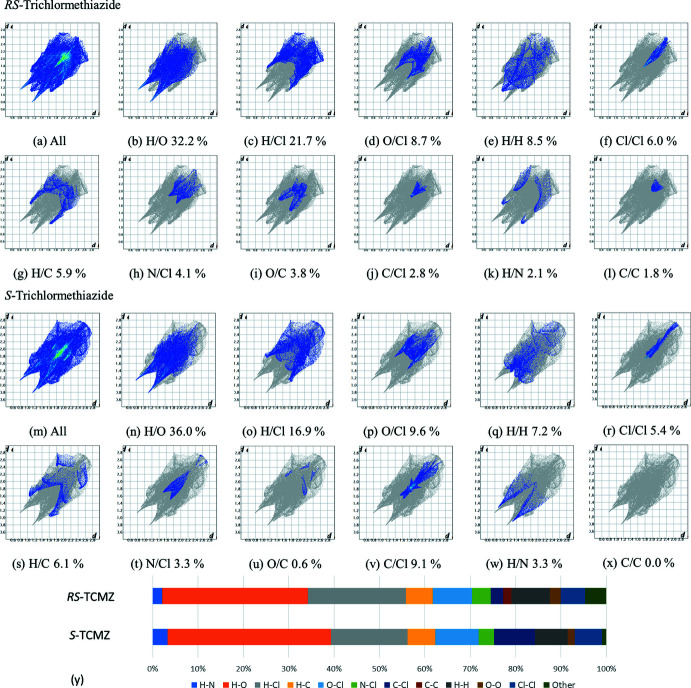
Fingerprint plots for *RS*-trichlorme­thia­zide: (*a*) all contacts; (*b*) H⋯O contacts; (*c*) H⋯Cl contacts; (*d*) O⋯Cl contacts; (*e*) H⋯H contacts; (*f*) Cl⋯Cl contacts; (*g*) H⋯C contacts, (*h*) N⋯Cl contacts; (*i*) O⋯C contacts; (*j*) C⋯Cl contacts; (*k*) H⋯N contacts; (*l*) C⋯C contacts. Fingerprint plots for *S*-Trichlorme­thia­zide: (*m*)–(*x*). The percentage of surface area included is shown for each plot: *RS*-TCMZ 95.3% and *S*-TCMZ 99%. (*y*) Comparison of the contribution of inter­molecular contacts (%) to the Hirshfeld surface area for *RS*-TCMZ and *S*-TCMZ.

Fingerprint plots representing *d*_e_/*d*_i_ inter­actions were calculated for *RS*-TCMZ and are shown in Fig. 8 ▸. For comparison, plots for *S*-TCMZ were also calculated and shown in the same figure, along with the contributions of all contacts in *RS*-TCMZ and *S*-TCMZ to the Hirshfeld surface area. As can be seen, there are significant differences between the fingerprint plots for both compounds. The full set of parameters calculated are presented in Fig. S3 of the additional supporting information.

Fig. 8 ▸ *a*–8*l* show that the most important inter­actions in *RS*-TCMZ are the H⋯O and H⋯Cl contacts, which represent 32.2 and 21.7% of the surface, respectively. In *S*-TCMZ, they are also the most important contacts (Fig. 8 ▸ *m*–8*x*) with 36.0 and 16.9%, respectively. The next inter­action, O⋯Cl, is slightly less important in *RS*-TCMZ than in *S*-TCMZ (8.7% *versus* 9.6%). The remaining inter­actions differ in order of importance. For example, the H⋯H inter­action is more important (8.5%) in *RS*-TCMZ than in *S*-TCMZ (7.2%). It is worth noting that the fingerprint plot delineated into the H⋯H inter­action for *RS*-TCMZ shows a tip at *d*_e_ + *d*_i_ = 2.20 Å, which is less than 2 times the van der Waals radii of hydrogen. In contrast, in *S*-TCMZ this inter­action is dispersed over a range of *d*_e_ + *d*_i_ values. Weaker inter­actions such as π–π contacts are present only in racemic TCMZ and they represent 1.8% of the contribution to the Hirshfeld surface. The Cl⋯π inter­action is more important in *S*-TCMZ, contributing 9.1% in contrast to *RS*-TCMZ where it represents 2.8%. This is the result of two inter­actions in the *S*-enanti­omer that lead to layers parallel to the *ab* plane. In *RS*-TCMZ, the Cl⋯π inter­actions alternate with π–π contacts to produce chains nearly along [101]. Another inter­esting feature is displayed by the H⋯N contacts. There is a lower degree of directionality and strength of this inter­action in *RS*-TCMZ (2.1%) than in *S*-TCMZ (3.3%) as a result of the additional N2—H2*A*⋯N1 inter­action in the latter.

**Figure 9:**
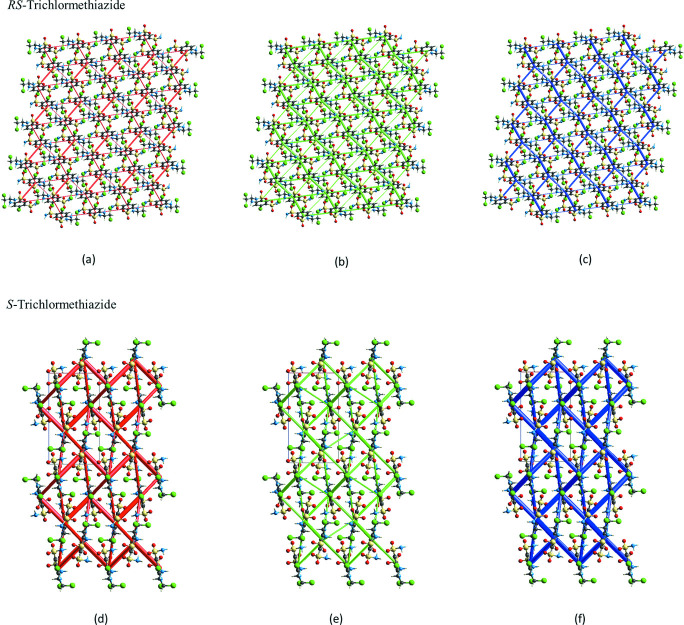
Energy frameworks calculated for *RS*-TCMZ viewed down the *b*-axis: (*a*) *E*_ele_, red; (*b*) *E*_dis_, green; (*c*) *E*_tot_, blue. Energy contributions are represented within 4 × 4 × 4 unit cells. The cylinder radii were scaled to 50 arbitrary units with a cut-off value of 10 kJ mol^−1^. Energy frameworks calculated for *S*-TCMZ viewed down the *b*-axis represented within 2 × 2 × 2 unit cells: (*d*) *E*_ele_, red; (*e*) *E*_dis_, green; and (*f*) *E_t_*_ot_, blue. The cylinder radii were scaled to 80 arbitrary units with a cut-off value of 5 kJ mol^−1^.

In addition, the electrostatic (*E*_ele_), dispersive (*E*_dis_), and total energies (*E*_tot_) for the inter­molecular inter­actions in *RS*-TCMZ and *S*-TCMZ were calculated with *CrystalExplorer21* (Spackman *et al.*, 2021 ▸). They are represented in Fig. 9 ▸. The summary of calculated energy values is presented in the supporting information and the detailed inter­actions are collected in Table S1 of the supporting information.

As depicted in Fig. 9 ▸, in *RS*-TCMZ the topologies of the electrostatic (*E*_ele_, Fig. 9 ▸ *a*) and dispersive (*E*_dis_, Fig. 9 ▸ *b*) components are similar although their contributions are quite different. They result in an offset tile arrangement for *E*_tot_ when viewed down the *b*-axis direction (Fig. 9 ▸ *c*). In the structure of *S*-TCMZ, *E*_ele_ and *E*_dis_ make similar contributions to *E*_tot_ and their topology is similar (Fig. 9 ▸ *d* and 9*e*). The pattern viewed down the *b*-axis direction resembles a herringbone arrangement (Fig. 9 ▸ *f*).

## Spectroscopic and thermal characterization

The FT–IR spectrum shows the absorption bands expected for TCMZ (Fig. S4 of the supporting information). The stretches for the secondary N—H grouping of the sulfonamide group appear at 3387 and 3322 cm^−1^ followed by the stretching bands of the S—N—H and N—H groups of the amine on the di­hydro­thia­diazine at 3281 and 3232 cm^−1^, respectively. The stretches of the C*sp* ^2^—H (3150–3100 cm^−1^) and C*sp* ^3^—H (3000–2900 cm^−1^) bonds are observed as weak bands. The C*sp* ^2^—C*sp* ^2^ stretch of the aromatic ring appears at 1596 cm^−1^ while the C—N and S—N stretches overlap at 1351 and 1332 cm^−1^. The stretches of the two S=O groups appear as strong absorptions at 1176 and 1157 cm^−1^. Table S2 summarizes the assignment of the most important absorptions for *RS*-TCMZ.

The TGA curve (Fig. S5*a*) recorded indicates the material is stable up to 240°C. A series of weight loss events occur from 240°C to 450°C. A 24.2% weight loss (2.270 mg) between 245 and 301°C coincides with the first two transitions in the DSC (Fig. S5*b*). The endotherm at 281.1°C (Δ*H* = 81.19 J g^−1^) is associated with melting of the material. This transition is followed by an exotherm with peak temperature 287.9°C (Δ*H* = 103.70 J g^−1^). The TGA curve shows two continuous weight loss processes at 302–384°C (1.354 mg, 14.4%) and 384–448°C (1.032 mg, 11.0%), associated with ill-defined transitions in the DSC. The total weight loss due to decomposition is 49.6%.

## Supplementary Material

Crystal structure: contains datablock(s) TCMZ, I. DOI: 10.1107/S2056989021013633/hb7999sup1.cif

Structure factors: contains datablock(s) I. DOI: 10.1107/S2056989021013633/hb7999Isup2.hkl

Supporting information file. DOI: 10.1107/S2056989021013633/hb7999Isup3.mol

Powder pattern superposition, Hydrogen bond propensity analysis, Hirshfeld surface, energy frameworks calculations, FT-IR, and TGA/DSC. DOI: 10.1107/S2056989021013633/hb7999sup4.pdf

Supporting information file. DOI: 10.1107/S2056989021013633/hb7999Isup5.cml

CCDC references: 2034096, 2034096

Additional supporting information:  crystallographic information; 3D view; checkCIF report

## Figures and Tables

**Table 1:** Geometry (Å, °) of hydrogen bonds, π–π, and C—Cl⋯π inter­actions in (*a*) *RS*-TCMZ and (*b*) S-TCMZ (KIKCUD) *Cg* is the centroid of the C1–C6 ring.

(*a*)					
*D*—H⋯*A*	*D*—H	H⋯*A*	*D*⋯*A*	*D*—H⋯*A*	Symmetry operation
N1—H1*A*⋯O4	0.950 (6)	2.024 (10)	2.874 (7)	148.0 (9)	−*x*, 2 − *y*, 1 − *z*
N1—H1*A*⋯O3	0.947 (7)	2.224 (8)	3.118 (7)	157.1 (6)	*x*, 1 + *y*, *z*
N2—H2*A*⋯O1	0.951 (7)	2.012 (10)	2.900 (7)	154.7 (10)	1 + *x*, *y*, *z*
N3—H3*A*⋯Cl3	0.952 (6)	2.767 (8)	3.108 (5)	102.1 (5)	
N3—H3*A*⋯O2	0.952 (6)	2.125 (7)	2.965 (6)	146.4 (8)	−*x*, 2 − *y*, −*z*
C6—H6⋯O1	0.953 (6)	2.372 (8)	2.801 (7)	106.9 (5)	
C7—H7⋯O4	0.953 (6)	2.584 (10)	2.991 (7)	106.1 (5)	
					
	*d*	α/β/γ	*CgI*_Perp/*CgJ*_Perp	Slippage	
*Cg*⋯*Cg* ^i^	4.401 (3)	0.0 (2)/26.2/26.2	3.9491 (19)/3.9487 (19)	1.942	−*x*, 2 − *y*, −*z*
	*X*⋯*Cg*	*X*-Perp	γ	*Y*—*X*⋯*Cg*	
C8—Cl2⋯*Cg* ^ii^	3.761 (5)	3.663	13.07	91.52 (19)	1 − *x*, 2 − *y*, 1 − *z*
					
(*b*)					
*D*—H⋯*A*	*D*—H	H⋯*A*	*D*⋯*A*	*D*—H⋯*A*	Symmetry operation
N1—H1*A*⋯O4	0.88 (3)	2.05 (3)	2.900 (4)	164 (3)	{1\over 2} + *x*, {1\over 2} − *y*, −*z*
N1—H1*B*⋯Cl1	0.88 (3)	2.75 (3)	3.353 (3)	127 (2)	
N1—H1*B*⋯O2	0.88 (3)	2.24 (3)	2.899 (4)	132 (3)	{1\over 2} + *x*, {3\over 2} − *y*, −*z*
N2—H2*A*⋯N1	0.88	2.30	3.127 (4)	156	{1\over 2} − *x*, 1 − *y*, {1\over 2} + *z*
N3—H3*A*⋯Cl3	0.91 (4)	2.72 (3)	3.119 (3)	108 (2)	
N3—H3*A*⋯O1	0.91 (4)	2.24 (4)	3.099 (4)	157 (3)	{1\over 2} + *x*, {1\over 2} − *y*, −*z*
C6—H6⋯O1	0.95	2.46	2.861 (4)	105	
C7—H7⋯O4	1.00	2.51	2.934 (4)	105	
C8—H8⋯O3	1.00	2.38	3.029 (4)	122	{1\over 2} − *x*, −*y*, {1\over 2} + *z*
	*X*⋯*Cg*	*X*-Perp	γ	*Y*—*X*⋯*Cg*	
C8—Cl2⋯*Cg* ^iii^	3.4556 (18)	−3.425	7.61	96.72 (12)	−*x*, −{1\over 2} + *y*, {1\over 2} − *z*

**Table 2:** Experimental details

Crystal data	
Chemical formula	C_8_H_8_Cl_3_N_3_O_4_S_2_
*M*_r_	380.64
Crystal system, space group	Triclinic, *P*\overline{1}
Temperature (K)	298
*a*, *b*, *c* (Å)	8.4389 (6), 8.8929 (7), 9.7293 (8)
α, β, γ (°)	91.315 (3), 106.113 (2), 97.1580 (17)
*V* (Å^3^)	694.73 (9)
*Z*	2
Radiation type	Cu *K*α_1_, λ = 1.5418 Å
Specimen shape, size (mm)	Flat sheet, 24.5 × 24.5

Data collection	
Diffractometer	Bruker D8 ADVANCE
Specimen mounting	Flat plate low-background Si single crystal specimen holder
Data collection mode	Reflection
Scan method	Step
2θ values (°)	2θ_min_ = 5.007, 2θ_max_ = 60.006, 2θ_step_ = 0.015

Refinement	
*R* factors and goodness of fit	*R*_p_ = 0.051, *R*_wp_ = 0.069, *R*_exp_ = 0.026, *R*_Bragg_ = 0.040, χ^2^ = 7.312
No. of parameters	177
No. of restraints	48
H-atom treatment	Only H-atom coordinates refined

Computer programs: *DIFFRAC.Suite* (Bruker, 2011 ▸), *TOPAS-Academic* (Coelho, 2018 ▸), *DASH* (David *et al.*, 2006 ▸), *DIAMOND* (Brandenburg, 1999 ▸), *enCIFer* (Allen *et al.*, 2004 ▸) and *publCIF* (Westrip, 2010 ▸).

## supplementary crystallographic information

### Crystal data

**Table:**

C_8_H_8_Cl_3_N_3_O_4_S_2_	*V* = 694.73 (9) Å^3^
*M**_r_* = 380.64	*Z* = 2
Triclinic, *P*1	*D*_x_ = 1.820 Mg m^−^^3^
Hall symbol: -P 1	Melting point: 554.25 K
*a* = 8.4389 (6) Å	Cu *K*α_1_ radiation, λ = 1.5418 Å
*b* = 8.8929 (7) Å	*T* = 298 K
*c* = 9.7293 (8) Å	Particle morphology: fine powder
α = 91.315 (3)°	white
β = 106.113 (2)°	flat_sheet, 24.5 × 24.5 mm
γ = 97.1580 (17)°	Specimen preparation: Prepared at 298 K and 100 kPa

### Data collection

**Table:**

Bruker D8 ADVANCE diffractometer	Data collection mode: reflection
Radiation source: sealed X-ray tube	Scan method: step
Specimen mounting: Flat plate low-background Si single crystal specimen holder	2θ_min_ = 5.007°, 2θ_max_ = 60.006°, 2θ_step_ = 0.015°

### Refinement

**Table:**

Least-squares matrix: full	48 restraints
*R*_p_ = 0.051	2 constraints
*R*_wp_ = 0.069	Only H-atom coordinates refined
*R*_exp_ = 0.026	Weighting scheme based on measured s.u.'s
*R*_Bragg_ = 0.040	(Δ/σ)_max_ = 0.001
3922 data points	Background function: Chebychev polynomial
Profile function: PseudoVoight	Preferred orientation correction: Simple March-Dollase correction, March-Dollase parameter:0.742(2)
177 parameters	

### Special details

**Table:**

Experimental. The FT-IR spectrum was registered in a IS50 FT-IR Nicolet Thermo Scientific spectrophotometer, in the 4000–400 cm^-1^ range with 32 scans at an optical speed of 0.4747 cm s^-1^. Thermogravimetric and differential scanning calorimetry measurements (TGA/DSC) were performed in a thermal analyzer DTA/DSC Instrument, Serie Discovery, under a dynamic nitrogen atmosphere at 50.0 mL min^-1^. The instrument was equilibrated at 28.00 °C. A heating ramp of 10.00 °C min^-1^ up to 450.00 °C was used.*RS*-Trichlormethiazide was gently ground in an agate mortar. Small portions of the material were dusted on top of a flat plate low-background Si single crystal specimen holder. Powder diffraction patterns were registered at room temperature on a Bruker D8 ADVANCE diffractometer working in the Bragg-Brentano geometry using CuKa radiation, operating at 40 kV and 30 mA, equipped with a LynxEye position-sensitive detector. The pattern used in the structure determination was recorded from 5.007 to 60.006° (2θ) in steps of 0.01526°, at 1.5 sec/step. The standard instrument settings (Ni filter of 0.02 mm, Soller slits of 2.5°, Divergence Slit of 0.2 mm, scatter screen height of 3 mm) were employed.
Geometry. Bond distances, angles etc. have been calculated using the rounded fractional coordinates. All su's are estimated from the variances of the (full) variance-covariance matrix. The cell esds are taken into account in the estimation of distances, angles and torsion angles

### Fractional atomic coordinates and isotropic or equivalent isotropic displacement parameters (Å^2^)

**Table:**

	*x*	*y*	*z*	*U*_iso_*/*U*_eq_	
Cl1	0.1706 (5)	1.3869 (4)	0.0737 (6)	0.077 (2)*	
Cl2	0.6976 (5)	0.6228 (5)	0.4823 (5)	0.077 (2)*	
Cl3	0.6169 (5)	0.6852 (6)	0.1807 (4)	0.077 (2)*	
S1	−0.1719 (4)	1.2122 (3)	0.1245 (3)	0.077 (2)*	
S2	0.1781 (3)	0.7747 (3)	0.3633 (3)	0.077 (2)*	
O1	−0.2933 (6)	1.0957 (6)	0.1498 (8)	0.067 (3)*	
O2	−0.1923 (8)	1.2685 (7)	−0.0171 (5)	0.067 (3)*	
O3	0.0321 (6)	0.6764 (7)	0.2898 (8)	0.067 (3)*	
O4	0.2180 (8)	0.8015 (8)	0.5171 (5)	0.067 (3)*	
N1	−0.1595 (5)	1.3476 (4)	0.2391 (3)	0.067 (3)*	
N2	0.4634 (3)	0.9538 (4)	0.2834 (4)	0.067 (3)*	
N3	0.3382 (4)	0.7092 (4)	0.3292 (3)	0.067 (3)*	
C1	0.0177 (3)	1.1394 (4)	0.1699 (5)	0.067 (3)*	
C2	0.1642 (4)	1.2130 (4)	0.1445 (6)	0.067 (3)*	
C3	0.3096 (3)	1.1530 (4)	0.1835 (5)	0.067 (3)*	
C4	0.3185 (3)	1.0161 (4)	0.2523 (5)	0.067 (3)*	
C5	0.1731 (4)	0.9465 (4)	0.2836 (5)	0.067 (3)*	
C6	0.0246 (3)	1.0070 (3)	0.2418 (5)	0.067 (3)*	
C7	0.4870 (3)	0.8221 (3)	0.3700 (3)	0.067 (3)*	
C8	0.6447 (4)	0.7593 (3)	0.3576 (3)	0.067 (3)*	
H1A	−0.1365 (14)	1.3166 (7)	0.3346 (5)	0.080 (3)*	
H1B	−0.1025 (9)	1.4428 (6)	0.2271 (6)	0.080 (3)*	
H2A	0.5578 (6)	1.0136 (7)	0.2680 (12)	0.080 (3)*	
H3	0.4081 (6)	1.2080 (7)	0.1708 (12)	0.080 (3)*	
H3A	0.3087 (8)	0.6781 (10)	0.2301 (5)	0.080 (3)*	
H6	−0.0758 (6)	0.9513 (7)	0.2507 (8)	0.080 (3)*	
H7	0.5057 (8)	0.8495 (7)	0.4690 (5)	0.080 (3)*	
H8	0.7352 (7)	0.8393 (6)	0.3793 (7)	0.080 (3)*	

### Geometric parameters (Å, º)

**Table:**

Cl1—C2	1.708 (6)	C1—C6	1.383 (5)
Cl2—C8	1.746 (5)	N1—H1B	0.947 (7)
Cl3—C8	1.771 (5)	N1—H1A	0.950 (6)
S1—O1	1.439 (6)	N2—H2A	0.951 (7)
S1—O2	1.449 (6)	C2—C3	1.360 (5)
S1—N1	1.596 (4)	C3—C4	1.403 (5)
S1—C1	1.747 (5)	N3—H3A	0.952 (6)
S2—O3	1.417 (7)	C4—C5	1.417 (5)
S2—O4	1.447 (5)	C5—C6	1.386 (5)
S2—N3	1.647 (4)	C7—C8	1.539 (4)
S2—C5	1.729 (5)	C3—H3	0.951 (7)
N2—C4	1.366 (4)	C6—H6	0.953 (6)
N2—C7	1.460 (4)	C7—H7	0.953 (6)
N3—C7	1.461 (4)	C8—H8	0.950 (7)
C1—C2	1.412 (5)		
			
O1—S1—O2	120.4 (4)	S2—N3—H3A	108.5 (5)
O1—S1—N1	104.7 (4)	C2—C3—C4	120.6 (3)
O1—S1—C1	106.3 (3)	N2—C4—C3	119.5 (3)
O2—S1—N1	110.6 (3)	N2—C4—C5	122.8 (3)
O2—S1—C1	106.4 (4)	C3—C4—C5	117.7 (3)
N1—S1—C1	107.9 (3)	C4—C5—C6	121.4 (3)
O3—S2—O4	119.4 (4)	S2—C5—C4	118.8 (3)
O3—S2—N3	108.0 (3)	S2—C5—C6	119.5 (3)
O3—S2—C5	108.2 (3)	C1—C6—C5	119.7 (3)
O4—S2—N3	107.2 (3)	N2—C7—N3	110.8 (3)
O4—S2—C5	109.4 (4)	N2—C7—C8	108.9 (2)
N3—S2—C5	103.4 (2)	N3—C7—C8	113.2 (2)
C4—N2—C7	122.0 (3)	Cl2—C8—Cl3	111.4 (3)
S2—N3—C7	111.5 (3)	Cl2—C8—C7	110.4 (3)
S1—C1—C2	123.5 (3)	Cl3—C8—C7	110.6 (3)
S1—C1—C6	117.3 (2)	C2—C3—H3	119.8 (6)
C2—C1—C6	119.1 (3)	C4—C3—H3	119.5 (6)
S1—N1—H1B	117.3 (5)	C1—C6—H6	119.4 (5)
H1A—N1—H1B	115.6 (7)	C5—C6—H6	120.4 (5)
S1—N1—H1A	113.0 (5)	N2—C7—H7	111.3 (5)
Cl1—C2—C1	121.7 (3)	N3—C7—H7	106.3 (5)
Cl1—C2—C3	116.8 (3)	C8—C7—H7	106.2 (5)
C4—N2—H2A	116.9 (5)	Cl2—C8—H8	107.1 (5)
C7—N2—H2A	119.3 (6)	Cl3—C8—H8	107.9 (5)
C1—C2—C3	121.5 (4)	C7—C8—H8	109.2 (4)
C7—N3—H3A	111.6 (6)		
			
O1—S1—C1—C2	172.5 (5)	S1—C1—C2—Cl1	3.2 (7)
O1—S1—C1—C6	−11.3 (5)	S1—C1—C2—C3	179.1 (4)
O2—S1—C1—C2	43.1 (5)	C6—C1—C2—Cl1	−172.9 (4)
O2—S1—C1—C6	−140.8 (4)	C6—C1—C2—C3	3.0 (7)
N1—S1—C1—C2	−75.6 (5)	S1—C1—C6—C5	−178.4 (4)
N1—S1—C1—C6	100.6 (4)	C2—C1—C6—C5	−2.0 (7)
O3—S2—N3—C7	165.6 (4)	Cl1—C2—C3—C4	175.2 (4)
O4—S2—N3—C7	−64.4 (4)	C1—C2—C3—C4	−0.8 (7)
C5—S2—N3—C7	51.2 (3)	C2—C3—C4—N2	176.4 (4)
O3—S2—C5—C4	−134.0 (4)	C2—C3—C4—C5	−2.1 (7)
O3—S2—C5—C6	41.0 (5)	N2—C4—C5—S2	−0.5 (6)
O4—S2—C5—C4	94.4 (5)	N2—C4—C5—C6	−175.4 (4)
O4—S2—C5—C6	−90.6 (5)	C3—C4—C5—S2	178.0 (4)
N3—S2—C5—C4	−19.6 (4)	C3—C4—C5—C6	3.1 (7)
N3—S2—C5—C6	155.4 (4)	S2—C5—C6—C1	−175.9 (4)
C7—N2—C4—C3	172.4 (4)	C4—C5—C6—C1	−1.0 (7)
C7—N2—C4—C5	−9.1 (6)	N2—C7—C8—Cl2	169.9 (3)
C4—N2—C7—N3	42.5 (5)	N2—C7—C8—Cl3	−66.3 (3)
C4—N2—C7—C8	167.7 (3)	N3—C7—C8—Cl2	−66.3 (3)
S2—N3—C7—N2	−64.5 (3)	N3—C7—C8—Cl3	57.5 (3)
S2—N3—C7—C8	172.8 (2)		

### Summary of calculated energies (kJ mol-1) for RS-TCMZ and S-TCMZ (from CSD Refcode KIKCUD)

**Table:**

	*E*_ele_	*E*_pol_	*E*_dis_	*E*_rep_	*E*_tot_
RS-TCMZ	-71.8	-50.6	-240.5	260.7	-161.6
S-TCMZ (KIKCUD)	-147.7	-41.4	-146.1	185.6	-199.6
					
Energy Model	——	*k*_ele_	*k_pol_*	*k*_disp_	*k*_rep_
CE-B3LYP B3LYP/6-31G(d,p) electron densities	——	1.057	0.740	0.871	0.618

### Predicted and realized intermolecular hydrogen bonds for TCMZ

**Table:**

Donor	Acceptor	Propensity	*RS*-TCMZ	*S*-TCMZ
Intermolecular interactions				
N1–H1B	O3	0.69	*Observed*	
N1–H1A	O4	0.69	*Observed*	*Observed*
N1–H1A	O1	0.68		
N1–H1B	O2	0.68		*Observed*
N3–H3A	O3	0.44		
N3–H3A	O4	0.44		
N3–H3A	O1	0.42		*Observed*
N3–H3A	O2	0.42	*Observed*	
N2–H2A	O3	0.42		
N2–H2A	O4	0.42		
N2–H2A	O1	0.41	*Observed*	
N2–H2A	O2	0.41		
N1–H1A/H1B	N2	0.08		
N1–H1A/H1B	Cl1	0.04		
N1–H1A/H1B	Cl2	0.04		
N1–H1A/H1B	Cl3	0.04		
N3–H3A	N2	0.03		
N2–H2A	N2	0.02		
N3–H3A	Cl1	0.02	*Observed*	
N2–H2A	Cl1	0.01		
N3–H3A	Cl2	0.01		
N3–H3A	Cl3	0.01		
N2–H2A	Cl2	0.01		
N2–H2A	Cl3	0.01		
Intramolecular interactions				
N1–H1B	Cl1	0.60		*Observed*
N3–H3A	Cl3	0.48	*Observed*	*Observed*
