# Supplementary material for: Crystal structure from X-ray powder diffraction data, DFT-D calculation, Hirshfeld surface analysis, and energy frameworks of (RS)-trichlorme­thia­zide
Source: Acta Crystallogr E Crystallogr Commun. 2022 Jan 7;78(Pt 2):140–8. doi: 10.1107/S2056989021013633 (PMC8819433; doi:10.1107/S2056989021013633)
Supplement: Supplementary file 4 [file e-78-00140-sup4.pdf]

SUPPLEMENTARY FIGURES AND TABLES FOR:

Crystal structure from X-ray powder diffraction data, DFT-D calculation, Hirshfeld surface analysis, and energy frameworks of racemic Trichlormethiazide

Robert A. Toro,[a] Analio Dugarte-Dugarte,[b] Jacco van de Streek,[c] José Antonio Henao,[a] José Miguel Delgado[b] and Graciela Díaz de Delgado[b*]

[a]Grupo de Investigación en Química Estructural (GIQUE), Escuela de Química, Facultad de Ciencias, Universidad Industrial de Santander, Bucaramanga, Colombia, [b]Laboratorio de Cristalografía-LNDRX, Departamento de Química, Facultad de Ciencias, Universidad de los Andes, Mérida 5101, Venezuela, and [c]Avant-garde Materials Simulation, Alte Str. 2, D-79249 Merzhausen, Germany.

Correspondence e-mail: gdiazdedelgado@gmail.com

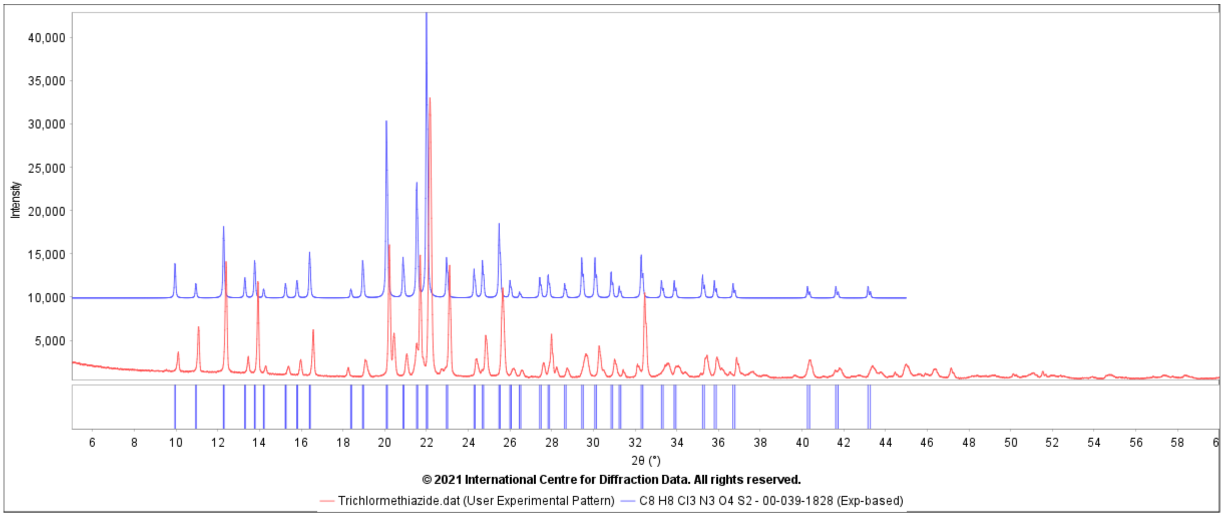

Figure S1. Superposition of the pattern recorded for *RS*-TCMZ with the pattern simulated using the data from entry PDF 00-039-1828.

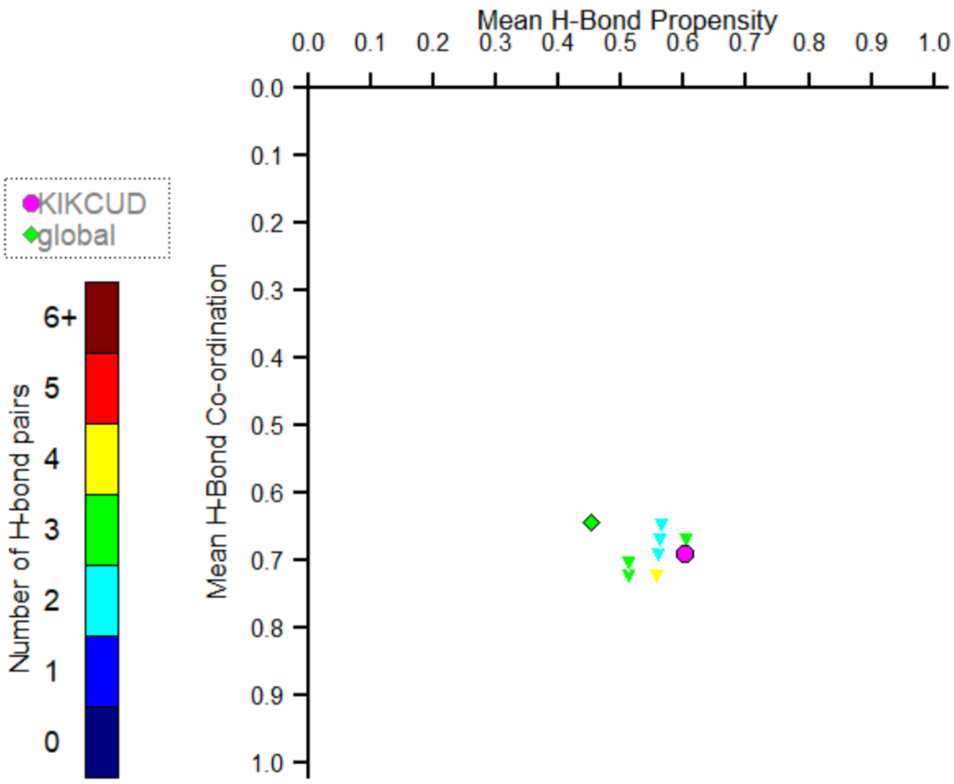

Figure S2. Putative landscape for trichlormethiazide. The experimental structures are highlighted, *RS*-TCMZ in green and *S*-TCMZ (KIKCUD) in pink.

RS-Trichlormethiazide

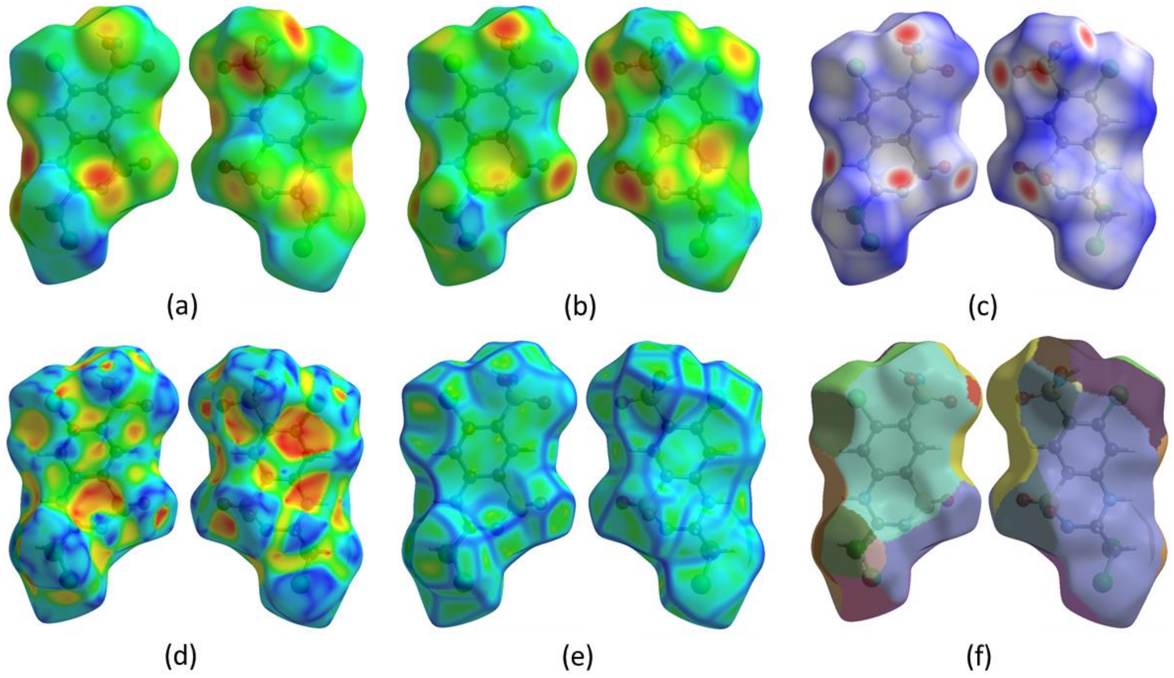

S-Trichlormethiazide

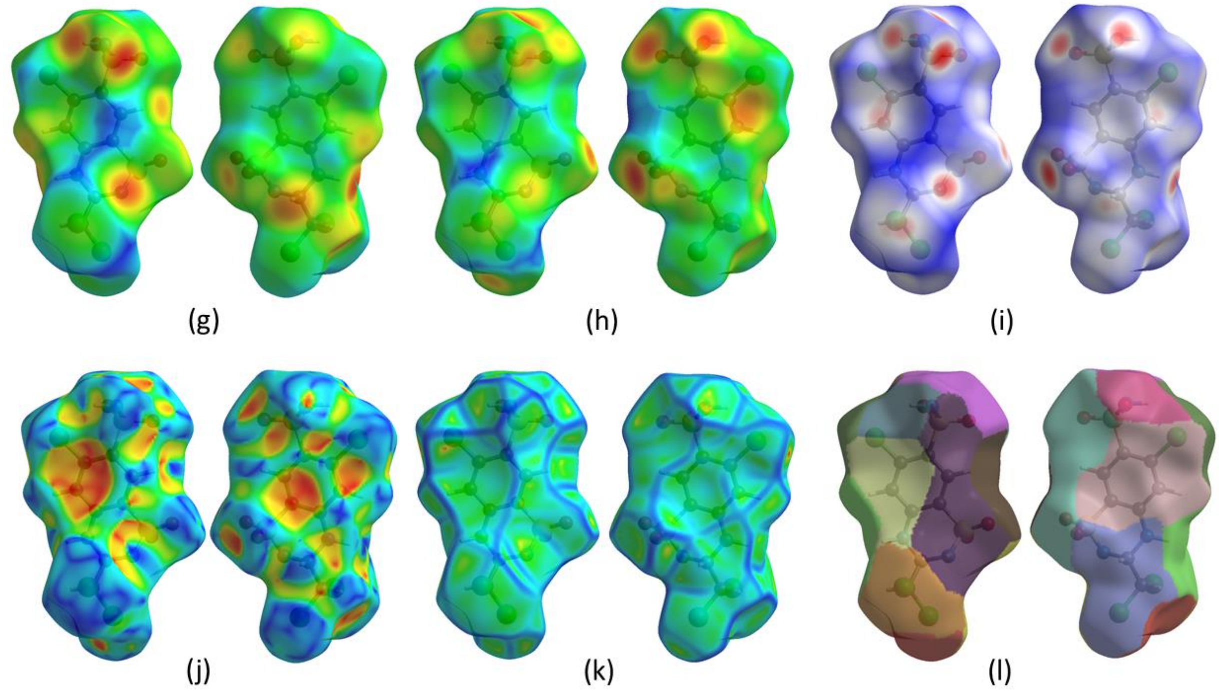

Figure S3. The parameters d_i, d_e, d_{norm}, shape index, curvedness, and fragment patch mapped onto the Hirshfeld surface for *RS*-TCMZ (a-f) and *S*-TCMZ (g-l).

Table S1. Interaction Energies (kJ/mol) for (a) *RS*-Trichlormethiazide and (b) *S*-Trichlormethiazide (KIKCUD).

- R is the distance between molecular centroids (mean atomic position) in Å.
- Total energies, only reported for two benchmarked energy models, are the sum of the four energy components, scaled appropriately (see the scale factor table below)

(a)	N	Symop	R	Electron Density	E_ele	E_pol	E_dis	E_rep	E_tot
	1	x, y, z	8.88	B3LYP/6-31G(d,p)	9.3	-1.4	-11.0	4.2	1.8
	1	-x, -y, -z	5.49	B3LYP/6-31G(d,p)	-19.9	-14.2	-58.8	46.9	-53.8
	1	-x, -y, -z	6.90	B3LYP/6-31G(d,p)	-32.7	-9.4	-22.3	37.7	-37.6
	2	x, y, z	8.43	B3LYP/6-31G(d,p)	-14.5	-11.5	-37.5	71.2	-12.5
	1	-x, -y, -z	11.41	B3LYP/6-31G(d,p)	-5.3	-2.0	-11.0	7.7	-11.9
	1	x, y, z	12.98	B3LYP/6-31G(d,p)	-1.1	-0.7	-3.5	1.0	-4.1
	0	-x, -y, -z	10.54	B3LYP/6-31G(d,p)	2.5	-3.6	-17.7	28.3	2.1
	1	-x, -y, -z	7.68	B3LYP/6-31G(d,p)	0.7	-1.4	-12.8	10.4	-5.0
	1	-x, -y, -z	5.56	B3LYP/6-31G(d,p)	-10.8	-6.4	-65.9	53.3	-40.6

(b)	N	Symop	R	Electron Density	E_ele	E_pol	E_dis	E_rep	E_tot
	2	x+1/2, -y+1/2, -z	6.26	B3LYP/6-31G(d,p)	-48.5	-12.4	-27.3	49.8	-53.5
	2	-x, y+1/2, -z+1/2	6.87	B3LYP/6-31G(d,p)	-32.9	-7.5	-38.5	27.4	-57.1
	2	x+1/2, -y+1/2, -z	11.21	B3LYP/6-31G(d,p)	-21.6	-4.9	-9.8	15.6	-25.5
	2	x, y, z	9.79	B3LYP/6-31G(d,p)	0.6	-2.0	-16.4	22.3	-1.4
	2	-x+1/2, -y, z+1/2	9.64	B3LYP/6-31G(d,p)	-18.7	-2.9	-15.9	20.2	-23.2
	2	-x, y+1/2, -z+1/2	8.13	B3LYP/6-31G(d,p)	-0.1	-1.5	-17.7	20.6	-3.8
	2	-x+1/2, -y, z+1/2	9.14	B3LYP/6-31G(d,p)	-26.5	-10.2	-20.5	29.7	-35.1

Energy Model	k_ele	k_pol	k_disp	k_rep
CE-HF ... HF/3-21G electron densities	1.019	0.651	0.901	0.811
CE-B3LYP ... B3LYP/6-31G(d,p) electron densities	1.057	0.740	0.871	0.618

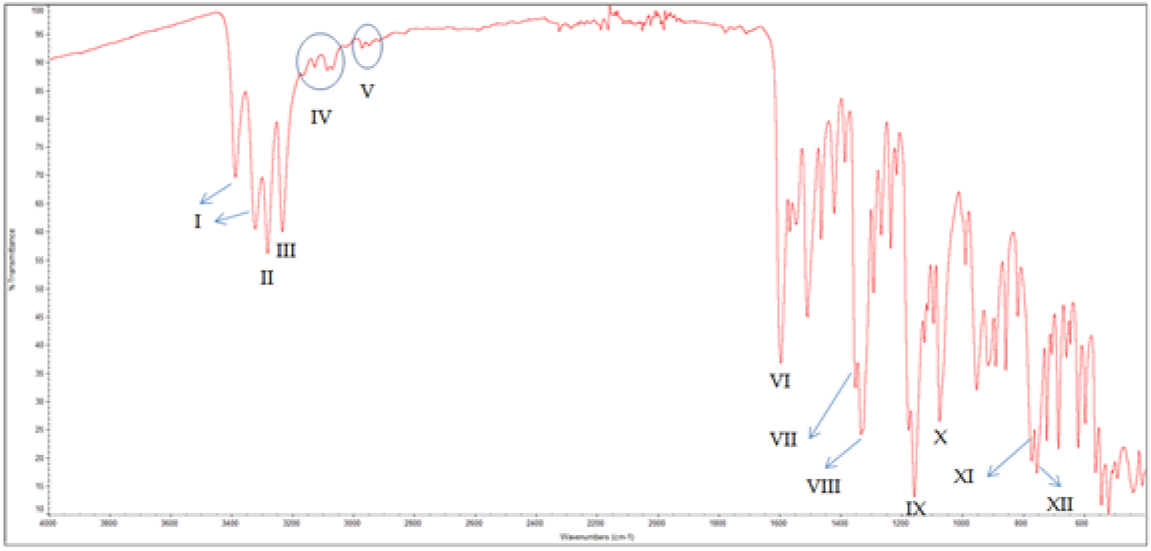

Figure S4. FT-IR of *RS*-Trichlormethiazide. The absorption bands assigned in Table S2 are indicated in Roman numerals.

Table S2. Assignments of main absorption bands for *RS*-Trichlormethiazide. Roman numerals correspond to the absorptions labeled in Figure S2.

**Absorption**	**Frequency (cm^{-1})**	**Assignment**
I	3386-3321.77	Stretch N-H (secondary)
II	3280.86	Stretch N-H (primary)
III	3232.09	Stretch N-H (primary amine)
IV	~3150-3100	Stretch –Csp^2-H
V	~3000-2900	Stretch –Csp^3-H
VI	1595.78	Stretch Csp^2-Csp^2 aromatic
VII	1350.69	Stretch C-N
VIII	1331.76	Stretch S-N
IX	1175.73-1157.32	Stretch S=O
X	1073.79	Deformation =C-S
XI	772.61	Deformation -C-S
XII	754.71	Stretch C-Cl

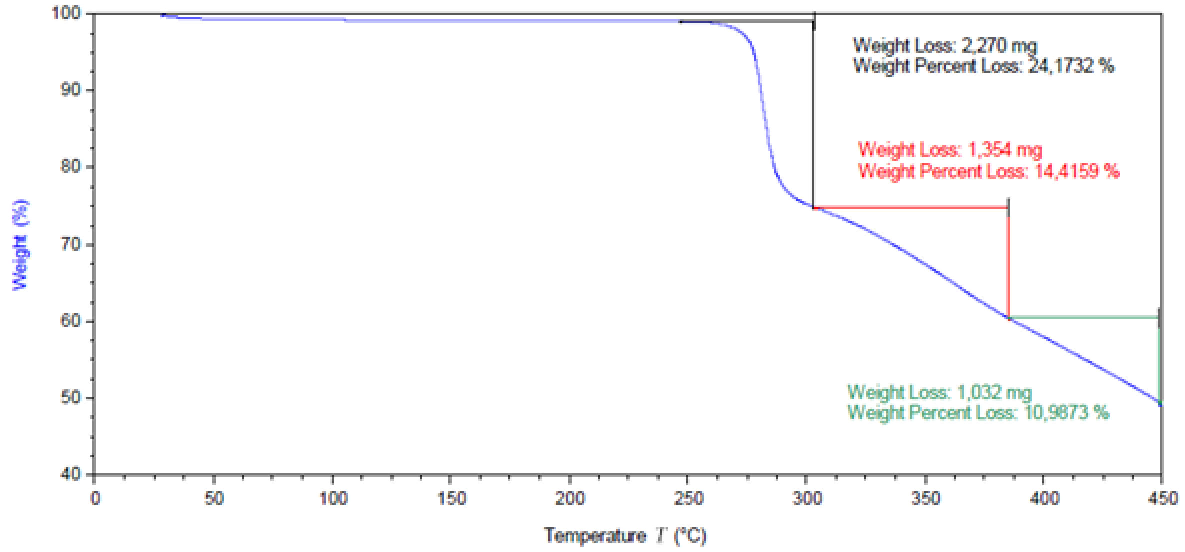

(a)

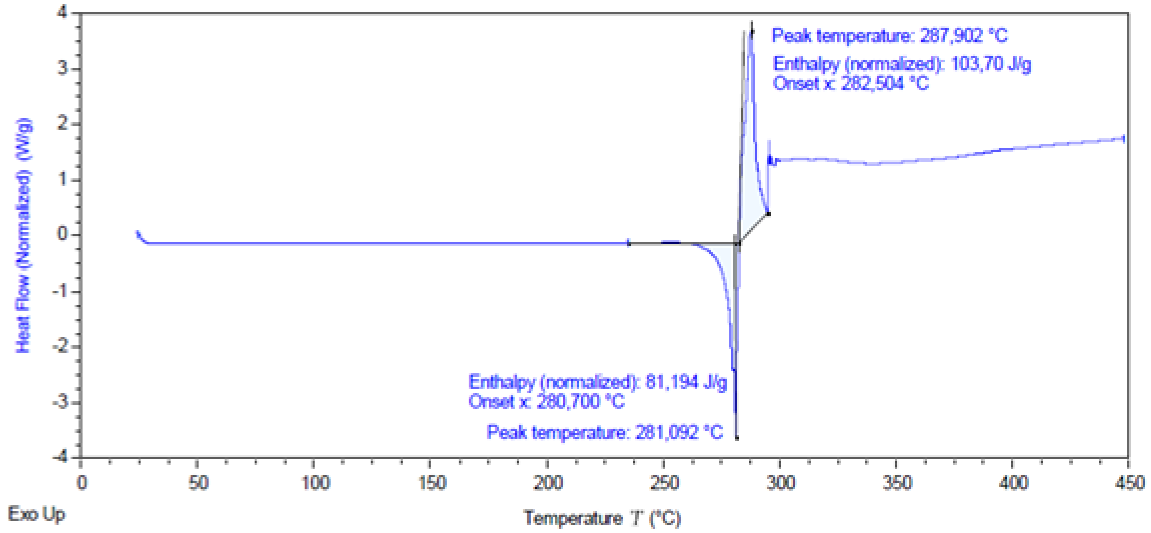

(b)

Figure S5. (a) TGA and (b) DSC curves for *RS*-Trichlormethiazide.
